# MR Volumetry of Lung Nodules: A Pilot Study

**DOI:** 10.3389/fmed.2019.00018

**Published:** 2019-02-12

**Authors:** Jean Delacoste, Vincent Dunet, Gael Dournes, Alban Lovis, Chantal Rohner, Christel Elandoy, Julien Simons, Olivier Long, Davide Piccini, Matthias Stuber, John O. Prior, Laurent Nicod, Catherine Beigelman-Aubry

**Affiliations:** ^1^Department of Radiology, Lausanne University Hospital (CHUV) and University of Lausanne, Lausanne, Switzerland; ^2^Centre de Recherche Cardio-Thoracique de Bordeaux, Université de Bordeaux, Bordeaux, France; ^3^Inserm, Centre de Recherche Cardio-Thoracique de Bordeaux, Bordeaux, France; ^4^CHU de Bordeaux, Service d'Imagerie Thoracique et Cardiovasculaire, Service des Maladies Respiratoires, Service d'Exploration Fonctionnelle Respiratoire, Pessac, France; ^5^Service of Pneumology, Department of Medicine, Lausanne University Hospital (CHUV), Lausanne, Switzerland; ^6^Department of Physiotherapy, Lausanne University Hospital (CHUV) and University of Lausanne, Lausanne, Switzerland; ^7^Advanced Clinical Imaging Technology, Siemens Healthcare AG, Lausanne, Switzerland; ^8^Center for Biomedical Imaging, Lausanne, Switzerland; ^9^Department of Nuclear Medicine and Molecular Imaging, Lausanne University Hospital, Lausanne, Switzerland

**Keywords:** lung, UTE, MRI, volumetry, segmentation, nodules

## Abstract

**Introduction:** Computed tomography (CT) is currently the reference modality for the detection and follow-up of pulmonary nodules. While 2D measurements are commonly used in clinical practice to assess growth, increasingly 3D volume measurements are being recommended. The goal of this pilot study was to evaluate preliminarily the capabilities of 3D MRI using ultra-short echo time for lung nodule volumetry, as it would provide a radiation-free modality for this task.

**Material and Methods:** Artificial nodules were manufactured out of Agar and measured using an ultra-short echo time MRI sequence. CT data were also acquired as a reference. Image segmentation was carried out using an algorithm based on signal intensity thresholding (SIT). For comparison purposes, we also performed manual slice by slice segmentation. Volumes obtained with MRI and CT were compared. Finally, the volumetry of a lung nodule was evaluated in one human subject in comparison with CT.

**Results:** Using the SIT technique, minimal bias was observed between CT and MRI across the entire range of volumes (2%) with limits of agreement below 14%. Comparison of manually segmented MRI and CT resulted in a larger bias (8%) and wider limits of agreement (−23% to 40%). *In vivo*, nodule volume differed of <16% between modalities with the SIT technique.

**Conclusion:** This pilot study showed very good concordance between CT and UTE-MRI to quantify lung nodule volumes, in both a phantom and human setting. Our results enhance the potential of MRI to quantify pulmonary nodule volume with similar performance to CT.

## Introduction

Thoracic computed tomography (CT) is the current reference imaging modality for the detection of nodules as well as for the follow-up of indeterminate pulmonary nodules incidentally discovered or occurring in a malignancy context. While 2D measurements are typically used in clinical practice, 3D volume measurements are growing in importance due to evidence that 3D volumetry is more robust and improves reproducibility (1). In the NELSON study, the use of CT volumetry diminished the need for follow-up evaluation in participants with an indeterminate test result. More precisely, the strategy entailed the use of the volume and volume-doubling time (VDT) for non-calcified indeterminate nodules between 50 and 500 mm^3^ (i.e., 4.6–9.8 mm in diameter). Growth was defined as an increase in volume of at least 25% between two scans (2), and a VDT <400 days was the main criteria suggestive of malignancy (3). The threshold of 25% was determined accounting for the substantial variation of semi-automated volume measurements of pulmonary nodules, the segmentation representing the most important factor contributing to measurement variability (4).

Although strategies for CT dose reduction have been applied, a radiation-free modality would be welcome for these tasks and the detection rate of lung nodules using MRI has been investigated in several studies. Good sensitivities for nodules of 4 mm and larger have been obtained owing to the development of new sequences improving image quality (5) with good signal and contrast-to-noise ratios, along with improved spatial resolutions (about 1 mm isotropic voxels) (6–10).

In a recent study, excellent performance of 3D radial ultra-short echo time (UTE) imaging at 3T was reported for the evaluation of lung nodules (11). A sensitivity of 92.4% was achieved for the detection of solid and subsolid nodules >4 mm, along with high diagnostic properties and interreader agreement for nodule morphologic assessment. However, UTE-MRI systematically underestimated dimension measurements by ~1–2 mm (11). According to the authors, if the tendency to systematically underestimate dimensions is taken into account when evaluating UTE-MRI studies, the bias could be compensated, potentially allowing UTE-MRI to substitute or complement thin-section CT for evaluating nodule size in routine clinical practice.

Despite the increased interest of volumetry with CT, no evaluation has, to our knowledge, been performed using MRI. In this preliminary work, we evaluated the feasibility of UTE-MRI volumetry in comparison with low-dose CT on artificial nodules and one *in vivo* data set.

## Materials and Methods

### MRI Segmentation Technique

A signal intensity threshold segmentation technique (12–14) was implemented to carry out segmentation on MRI images with Slicer 4.8.1 (http://www.slicer.org) (15) as follows: First, a spherical region of interest (ROI) was manually defined around each nodule. The ROI size was chosen so as to include the entire nodule plus an adequate margin of air around it. Within the ROI, the maximum (*I*_max_) and minimum (*I*_min_) voxel intensity were recorded. All voxels with signal intensity between *I*_max_ and *C* · (*I*_max_− *I*_min_)+ *I*_min_ were then considered part of the nodule. C is a variable percentage threshold which adjusts the lower bound of signal intensity for included voxels. With this equation, if C is equal to 0% all voxels within the ROI will be selected. As the value of C is increased, voxels with lower signal intensities will be discarded from the selection.

### Phantom Study

Artificial nodules were manufactured out of Agar (30 g/L) (16) by micropipetting different quantities of hot Agar into a mold fabricated from the hemispherically shaped part of a plastic pipette reservoir. After cooling, each artificial nodule was removed from the mold and weighed on a precision scale. The weight was then divided by the density of the Agar preparation to obtain the true volume of each artificial nodule. Twenty-two artificial nodules with a range of volumes between 16 and 561 mm^3^ were manufactured.

Imaging was performed within 4 h after manufacturing the artificial nodules. For imaging, the artificial nodules were placed between two layers of packing foam, which provided many interfaces with air to mimic lung parenchyma. A large cylindrical phantom with two compartments of agar was placed on top of the setup to provide sufficient MRI coil loading.

MRI acquisition was carried out on a 3T scanner (MAGNETOM Prisma, Siemens Healthcare, Erlangen, Germany) using a prototype double echo UTE sequence (17, 18) with spiral phyllotaxis trajectory (19). The following acquisition parameters were used: echo time (TE_1_) = 0.08 ms, TE_2_ = 2.86 ms, TR = 5.9 ms, readout bandwidth_1_ = 305 Hz/pixel, readout bandwidth_2_ = 610 Hz/pixel, radio-frequency excitation angle = 5°, field of view = (500 mm)^3^, matrix size = 512^3^, voxel size = (0.98 mm)^3^, and 1,220 segments consisting of 50 readouts each. Images were reconstructed using non-uniform fast Fourier transform.

CT acquisition was performed on a clinical scanner (Discovery 750 HD, GE Healthcare, Chicago, United States) using a low-dose protocol (computed tomography dose index of 2) with 1.25 mm thick overlapped slices reconstructed with soft kernel, as recommended for accurate segmentation (20).

Nodules were segmented on MRI images using the signal intensity threshold algorithm outlined above. Multiple values were compared for the threshold C (20, 30, 40, and 50%) to determine the one which results in the highest concordance with the known volume. In addition, segmentation was also carried out on MRI images by a chest imaging expert who traced manually the outline of each artificial nodule on each slice using the “Live Wire” mode of the “Lesion Management” module of our PACS system (Carestream, Rochester, New York, USA). CT image segmentation was carried out using the automatic CT lung lesion segmentation tool from the same module.

### *In vivo* Feasibility Study

We also compared the CT and UTE-MR volumetric assessment of a real nodule incidentally detected in the upper part of the right upper lobe of a volunteer from a recent study that explored a new technique allowing prolonged apnea-like MR acquisition performed at full inspiration (21). UTE acquisition was carried out during a 6-min long respiratory stabilization period. This study was carried out in accordance with the recommendations of the “Commission cantonale (VD) d'éthique de la recherche sur l'être humain” with written informed consent from all subjects. All subjects gave written informed consent in accordance with the Declaration of Helsinki. The protocol was approved by the “Commission cantonale (VD) d'éthique de la recherche sur l'être humain.” UTE-MR volumetry was carried out with the same procedure as for artificial nodules. For the signal intensity thresholding, the value of C that gave the highest concordance with CT *in vitro* was used. Nodule volume was assessed on CT with the software used in routine practice in our PACS system by using the lung and soft kernel, the latter being recommended for accurate segmentation (20). Additionally, average diameter measurement were carried out, as recommended by the Fleischner Society as the average of long and short axes more accurately reflects three-dimensional tumor volume (8).

### Statistical Analysis

As in the NELSON study volume doubling times were calculated only for nodules with volumes>50 mm^3^, we explicitly excluded the four artificial nodules with volume<50 mm^3^ from analysis, as they would not require a volume doubling time assessment at this size. X-Y plots were used to compare volume measurements obtained from phantom MRI data to the values derived from weight measurements and the ones resulting from CT image segmentation. Lin's concordance analysis was carried out to quantify the relationships. In addition percent-based Bland-Altman analysis was performed on measurements obtained with the value of C offering the best correlation to assess the bias and limits of agreement between MRI and CT in phantoms.

## Results

### Phantom Study

**Figure 1:**
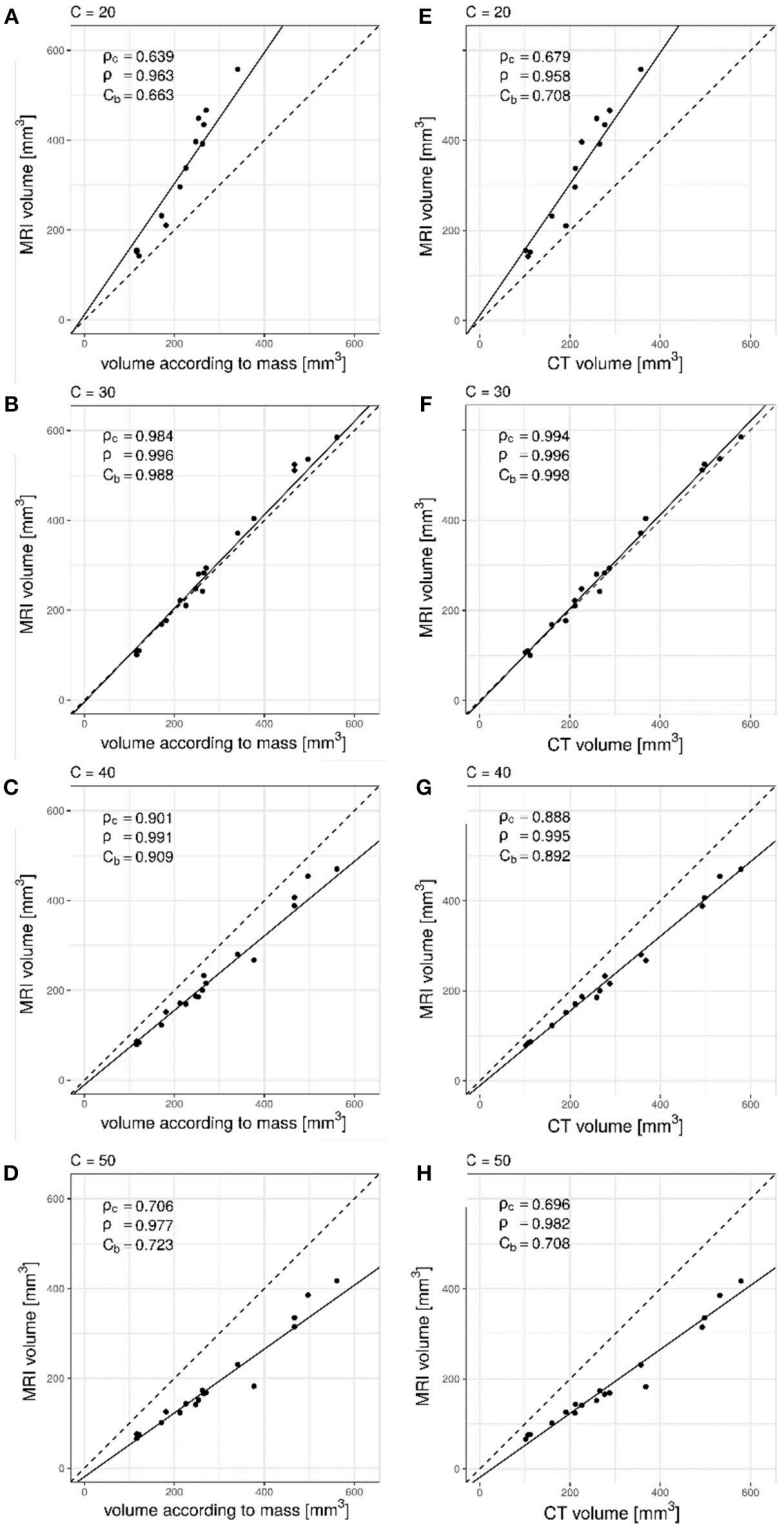
Lin's concordance analysis between artificial nodule volumes measured on UTE MRI data relative to mass derived volume measurements for increasing values of C. Dashed line is the line of equality. Solid line indicates reduced major axis linear regression. **(A)** C = 20%, **(B)** C = 30%, **(C)** C = 40%, **(D)** C = 50%. Pearson correlation (ρ) was larger than 0.9 with all values while the bias correction factor (*C*_*b*_) varied between 0.663 and 0.988. The deviation from the line of equality could also be appreciated. Comparison between CT and UTE MRI showed similar results, as seen in **(E)** C = 20%, **(F)** C = 30%, **(G)** C = 40%, and **(H)** C = 50%. Excellent precision (ρ = 0.996) and minimal bias between measurement methods (*C*_*b*_ = 0.998) was observed for a value of C = 30%.

When comparing MRI volume measurements to the volume according to the mass of the artificial nodules (Figures 1A–D) or with CT based volume measurements (Figures 1E,F), in all cases the Pearson correlation coefficient (ρ) was larger than 0.9. However, the bias correction factor (*C*_*b*_) varied between 0.663 and 0.998 depending on the value used for the signal intensity threshold C. The highest value was obtained with a threshold C equal to 30%. The best concordance in terms of Lin's coefficient (ρ_*c*_) was also obtained with a threshold for MRI segmentation equal to 30%.

**Figure 2:**
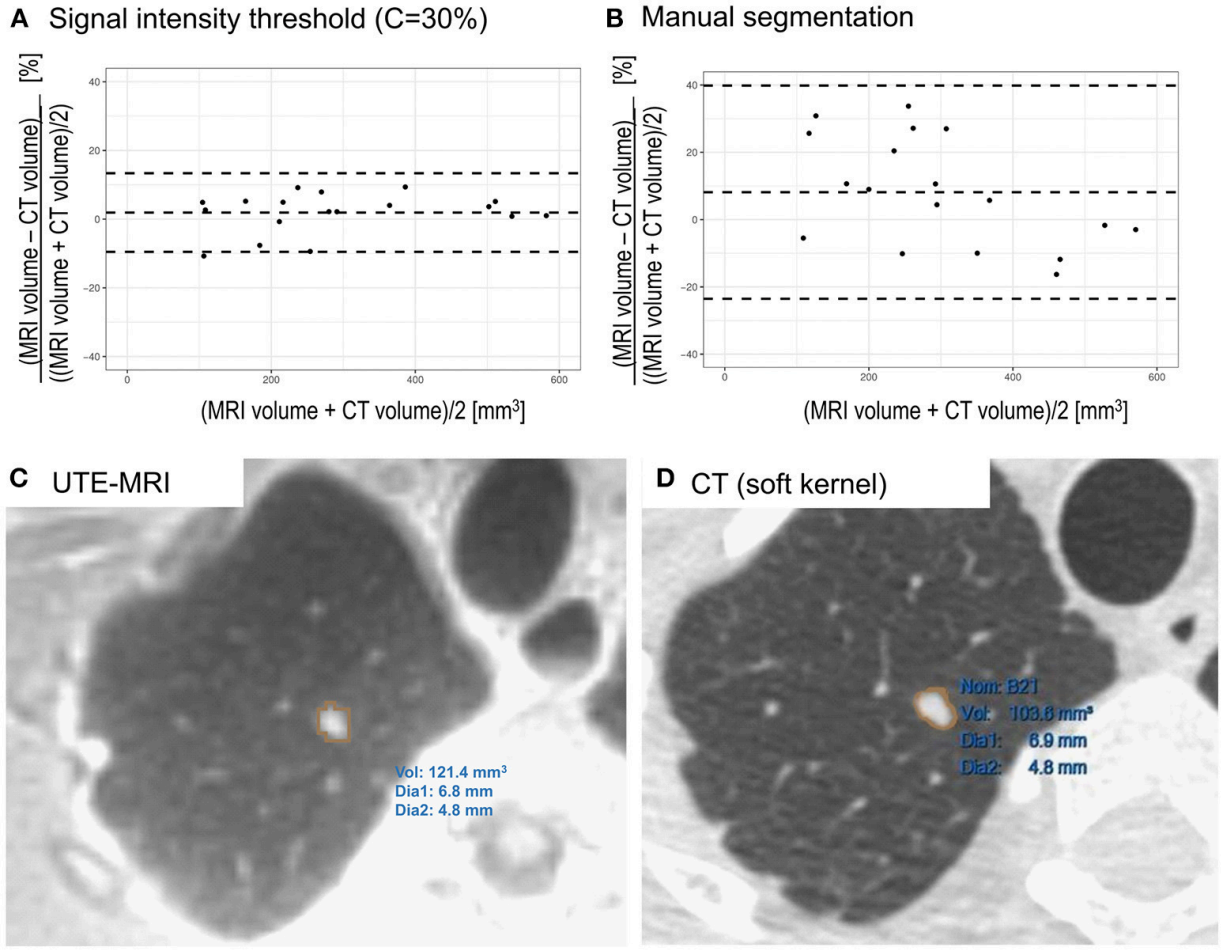
**(A)** Bland-Altman plot comparing CT and UTE MRI (signal intensity threshold C = 30%) based volume measurements of artificial nodules. Differences are expressed as percentage. Minimal bias was observed across the entire range of volumes (2%) with limits of agreement below 14% (−10% to 14%). **(B)** Comparison of CT and manually segmented MRI of artificial lung nodules. A slightly larger bias (8%) was observed and limits of agreement were wider (−23% to 40%). **(C)** Segmentation of lung nodule in a volunteer on UTE MRI image using threshold based segmentation in Slicer. **(D)** Segmentation of the same lung nodule on CT image reconstructed with soft kernel using semi-automatic segmentation tool.

Bland-Altman analysis between CT and MRI, using signal intensity threshold segmentation (C = 30%) indicated limits of agreement below 14% (Figure 2A) with minimal bias (2%) from the smallest to the largest artificial nodule volumes. When manual segmentation was used (Figure 2B), a slightly larger bias (8%) was observed and limits of agreement were wider (−23 to 40%) with respect to CT volume.

### *In vivo* Feasibility Study

On *in vivo* images, the nodule could be successfully segmented in MRI data using the investigated signal intensity threshold technique (Figure 2C). The volume was determined to be 121 mm^3^, with an average diameter of 6 mm. On CT images, when using the lung kernel, a volume of 110 mm^3^ was obtained while with the soft kernel images a value of 104 mm^3^ (Figure 2D) was reached. In both cases, the average diameter measured was of 6 mm. Hence, the differences in volumes were of 10% (lung kernel) and 16% (soft kernel) between CT and MRI segmented using signal intensity threshold. Manual slice by slice segmentation of the MRI resulted in a larger volume measurement (146 mm^3^), with an average diameter of 6 mm. With this manual segmentation, the differences between CT and MRI were between 33% (lung kernel) and 40% (soft kernel).

## Discussion

While demonstrating that MR pulmonary nodule volumetry is feasible, we observed an inter-modality difference of <20% both *ex-vivo* and *in-vivo* with signal intensity threshold segmentation when using a parameter C of 30%. This difference, both in phantoms and *in vivo*, remains below the threshold of 25% that defines a change in nodule volume between two examinations (2). Therefore, our results suggest that 3D-UTE-MRI could be used as an alternative to low-dose 3D-CT for pulmonary nodule volume evaluation. This could drastically reduce population exposure to CT related irradiation.

With the lowest signal intensity threshold C = 20% the volume measured in phantoms was overestimated, as could be appreciated graphically (Figures 1A,E). Values of 40% and 50% on the other hand resulted in underestimation of the volume (Figures 1C,D,G,H) as not enough lower intensity voxels were included in the segmentation. The optimal value was found to be 30% (Figures 1B,F) as it resulted in excellent correlation (ρ_*c*_ = 0.984 and ρ_*c*_ = 0.994), precision (ρ = 0.996 and ρ = 0.996) as well as minimal bias (*C*_*b*_ = 0.988 and *C*_*b*_ = 0.998).

While artificial nodules were surrounded by foam, the background signal in true lung parenchyma is expected to be higher. This could potentially make segmentation more difficult as the signal transition may be less well-defined between nodules and the surrounding parenchyma. However, with the *in vivo* case evaluated in this feasibility study, volumetry was still consistent between CT and MRI despite the presence of such parenchymal signal. To generalize this finding, the robustness of the method needs confirmation on a large cohort including both solid and subsolid nodules. This work is still ongoing in our institution. The concordance of volume doubling time across modalities should also be investigated. Furthermore, the potential influence of gadolinium injection also requires further assessment. The impact of the MR signal intensity threshold (i.e., 30%) on volumetric variability across MR manufacturers or UTE variants should also be evaluated since it could be a limitation in multicenter studies.

The only manual step in the signal intensity threshold method was the placement of the ROI within which the thresholding operation should be performed. The reduced operator dependence should result in high reproducibility of measurements but future investigations are required to ascertain this, especially *in vivo*. In phantoms, manual delineation of nodule outlines slice by slice was challenging, as due to partial volume effects and the texture of the nodules, the boundaries were not clearly defined. This resulted in larger bias and limits of agreement (Figure 2B) than the objective signal intensity threshold segmentation technique (Figure 2A). The procedure studied in this work should also be evaluated using commercial software from different vendors that also generally offer threshold segmentation tools. If differences of volumes measured across platforms remain limited, widespread clinical use could be enabled.

In our current setting, MRI examination, including patient installation and image acquisition with a single UTE acquisition, may be carried out in under 30 min. While this is longer than the time required for a standard CT protocol (~10 min), the duration remains reasonable for clinical workflow. Both CT and MRI segmentation took <5 min.

A limitation of this study is that only a single, incidentally discovered, *in vivo* nodule was available for study. Further studies are required to explore the impact of nodule texture and position on MRI volume measurements. In particular, the reliability of volume measurements close to the lung bases needs confirmation. Although the respiratory stabilization method used (21) greatly diminishes motion compared to free-breathing acquisitions, residual motion artifacts may remain, particularly at the lung bases. Related to this issue, it also remains to be ascertained that respiratory stabilization can also be reliably achieved in patients with lung disease.

In conclusion, this first study shows very good concordance between CT and UTE-MRI to quantify lung nodule volumes, in both a phantom and human conditions. In addition to the already known capabilities of MR to characterize tissue contrasts, our results enhance the potential of high resolution MRI to also quantify pulmonary nodule volume with similar performance to that of CT. Future assessment in a larger cohort may eventually promote MRI as a powerful and reproducible tool for lung nodule management, especially in the setting of indeterminate nodules that would require repetition of imaging and/or more specific evaluation, in a radiation-free manner.

## Author Contributions

JD, VD, GD, AL, MS, JP, LN, and CB-A contributed conception and design of the study. JD, AL, CR, CE, JS, OL, and DP carried out preparation of samples and performed image acquisition. JD, VD, GD, and CB-A contributed to image segmentation and analysis. JD performed statistical analysis. JD, GD, and CB-A wrote the first draft of the manuscript. All authors contributed to manuscript revision, read and approved the submitted version.

### Conflict of Interest Statement

JD reports personal fees from Swiss National Science Foundation and non-financial support from Siemens Healthineers, during the conduct of the study. MS reports grants from Swiss National Science Foundation and non-financial support from Siemens Healthineers, during the conduct of the study. AL reports non-financial support from Acutronic, during the conduct of the study. CB-A reports grants from Swiss National Science Foundation and non-financial support from Siemens Healthineers, during the conduct of the study; personal fees from Gilead, outside the submitted work. The remaining authors declare that the research was conducted in the absence of any commercial or financial relationships that could be construed as a potential conflict of interest.
